# Obstructive sleep apnea in Norwegian adults with achondroplasia: a population-based study

**DOI:** 10.1186/s13023-021-01792-7

**Published:** 2021-04-07

**Authors:** Svein O. Fredwall, Britt Øverland, Hanne Berdal, Søren Berg, Harald Weedon-Fekjær, Ingeborg B. Lidal, Ravi Savarirayan, Grethe Månum

**Affiliations:** 1 TRS National Resource Centre for Rare Disorders, Sunnaas Rehabilitation Hospital, 1450 Nesodden, Norway; 2 Faculty of Medicine, Institute of Clinical Medicine, University of Oslo, Oslo, Norway; 3 Department of Sleep Disorders, Lovisenberg Diakonale Hospital, Oslo, Norway; 4 Research Support Service, Oslo Centre for Biostatistics and Epidemiology, Oslo University Hospital, Oslo, Norway; 5 Murdoch Children’s Research Institute and University of Melbourne, Parkville, Australia; 6 Department of Research, Sunnaas Rehabilitation Hospital, Nesodden, Norway

**Keywords:** Obstructive sleep apnea, Sleep-disordered breathing, Hypertension, Body mass index, Craniofacial abnormalities

## Abstract

**Background:**

Previous studies have found a high prevalence of obstructive sleep apnea (OSA) in children with achondroplasia, but clinical studies on this complication in adults with achondroplasia are lacking.

**Objectives:**

This population-based, cross-sectional study investigated the prevalence, severity, and predictive factors of OSA in Norwegian adults with achondroplasia.

**Methods:**

We collected clinical data on 49 participants. Participants without a preexisting diagnosis of OSA had an overnight sleep registration. OSA was defined as an apnea–hypopnea index (AHI) ≥ 5 plus characteristic clinical symptoms, or AHI ≥ 15. We used the Berlin Questionnaire to assess clinical symptoms of OSA.

**Results:**

OSA was found in 59% (29/49) of the participants (95% confidence interval 44 to 73%), of whom 59% (17/29) had moderate to severe OSA (AHI ≥ 15), and 48% (14/29) were previously undiagnosed. Variables predictive of OSA were: excessive daytime sleepiness; unrested sleep; loud snoring; observed nocturnal breathing stops; hypertension; age > 40 years; and BMI > 30 kg/m^2^.

**Conclusion:**

OSA was highly prevalent in Norwegian adults with achondroplasia, which we believe is representative of this population worldwide. Follow-up of adults with achondroplasia should include assessment of symptoms and signs of OSA, with a low threshold for conducting an overnight sleep registration if findings suggestive of OSA are present.

## Background

Achondroplasia is the most common skeletal dysplasia, with an estimated prevalence of about 1: 25,000–30,000 [1]. The condition is caused by a gain-of-function mutation in the fibroblast growth factor receptor 3 (*FGFR3*) gene, resulting in disturbed bone growth, affecting the long bones, the spine, and the skull [1, 2]. Characteristic features are marked short stature, in particular short extremities (rhizomelia), macrocephaly, frontal bossing and midface hypoplasia [1]. In infants, foramen magnum stenosis is the most severe complication, potentially causing compression of the brain stem, hydrocephalus, central sleep apnea and sudden death [1, 3, 4]. Individuals with achondroplasia are also predisposed for developing obesity [5–7].

OSA is a breathing disorder characterized by narrowing of the upper airway that impairs normal ventilation during sleep [8]. Characteristic symptoms of OSA are excessive daytime sleepiness, unrested sleep, loud disruptive snoring, and observed nocturnal breathing stops, choking or gasping [9, 10]. The consequences of undiagnosed or untreated OSA can be severe, including hypertension, increased risk of cardiovascular disease, metabolic disorders, stroke, and traffic and workplace accidents [10–12]. In average-statured adults, obesity (in particular abdominal obesity), craniofacial abnormalities, male sex, age between 40 and 70 years, and smoking, are well-known risk factors for OSA [9–11].

Several studies have reported a high prevalence (often 50% or higher) of obstructive sleep apnea (OSA) in children with achondroplasia [13–15], compared to 1–4% prevalence in average-statured children [12, 16]. In the average-statured adult population, the estimated prevalence of OSA is 4–6% when OSA is defined as an apnea–hypopnea index (AHI) of ≥ 15, or an AHI ≥ 5 plus characteristic symptoms [9, 12]. We are not aware of previous clinical studies investigating prevalence and severity of OSA in adults with achondroplasia [17].

Enlarged tonsils and/or adenoids are common in achondroplasia, and for children diagnosed with OSA tonsillectomy and/or adenotonsillectomy is the recommended first-line treatment [13, 14, 18, 19]. However, previous studies have shown that OSA may persist after upper airway surgery [18, 20, 21]. Assessment by polysomnography is now recommended as part of routine follow-up in infants and children with achondroplasia [13, 22, 23], but there are currently no specific recommendations for assessing OSA in adults.

The objectives of this study were to investigate the prevalence and severity of OSA in Norwegian adults with achondroplasia, including clinical variables predictive of OSA in this condition.

## Methods

### Study design, population and data collection

This cross-sectional study was part of The Norwegian Adult Achondroplasia Study, a population-based study conducted between 2017 and 2019 on community-dwelling, Caucasian adults, aged 16 years or older, living in Norway [24]. Details of the recruitment process, and inclusion and exclusion criteria in The Norwegian Adult Achondroplasia Study have been described elsewhere [24].

The investigations were conducted during a 2.5-day stay at Sunnaas Rehabilitation Hospital. Medical history was obtained in a face-to-face interview, and included preexisting diagnosis of OSA, history of upper airway surgery, current medication or treatment of OSA, history of hypertension, and smoking habits.

### Definition of OSA

According to the International Classification of Sleep Disorders (3^rd^ edition), apnea was defined as ≥ 90% reduction of airflow from baseline for ≥ 10 s, and hypopnea as ≥ 30% reduction of airflow from baseline for ≥ 10 s combined with an oxygen desaturation of ≥ 3% [25, 26]. The diagnosis of OSA required either ≥ 5 predominantly obstructive respiratory events per hour plus characteristic symptoms of OSA, or ≥ 15 obstructive respiratory events per hour. Characteristic symptoms were excessive daytime sleepiness, restless sleep, loud snoring, observed nocturnal breathing stops, choking or gasping, or presence of hypertension [26]. The severity of OSA was defined as mild (AHI 5–14.9), moderate (AHI 15–29.9) or severe (AHI ≥ 30) [8].

### Clinical measurements

Height was measured in centimeters (cm), using a wall-mounted measuring tape. Weight was measured in kilograms (kg) using a digital weight. Body mass index (BMI) was calculated as weight divided by height squared. Blood pressure was measured in the morning, using a digital blood pressure monitor with a small cuff [7]. Hypertension was defined according to the European Society of Cardiology’s guidelines (2018) as either systolic blood pressure ≥ 140 mm Hg or diastolic blood pressure ≥ 90 mm Hg [27], or antihypertensive drug treatment.

### Sleep registration

The American Academy of Sleep Medicine recommends polysomnography, or home sleep monitoring with an adequate device, to diagnose OSA [8]. We used the NOX T3™ (NOX Medical Global, Reykjavik, Iceland), a widely used type 3 portable sleep monitor, validated and well-documented for diagnosing OSA in adults [28]. The NOX T3 provides measurements of nasal airflow, chest and abdominal movements, oxygen saturation (pulse oximetry), heart rate, and body position (actigraphy).

All participants in this study, except those with a preexisting diagnosis of OSA, had a single-night, unattended, sleep registration with the NOX T3 during the hospital stay. Medical staff, experienced with the equipment, conducted the patient hook-up at bedtime. The participants were instructed to make a notice of the time they went to sleep, and the time they woke up the next morning. We downloaded and pre-reviewed the sleep records for technical acceptability the following morning. We required a minimum of 4 h of recording time with acceptable quality. Participants were asked to undergo a second registration the following night if the first sleep record was technically unacceptable. An experienced sleep physiologist (BØ) manually examined and scored the sleep records. For those with a preexisting diagnosis of OSA, we collected previous sleep records to confirm the diagnosis of OSA.

### Berlin questionnaire

The Berlin Questionnaire (BQ) is a widely used screening tool to classify patients as high or low risk of OSA [8, 29, 30]. The BQ consists of 11 items, divided into three categories: (1) snoring, (2) daytime somnolence, and (3) presence of hypertension or obesity (BMI > 30 kg/m^2^) [31]. The BQ has been translated into Norwegian, and validated for the Norwegian general population [30]. In this study, we used the BQ to assess clinical symptoms of OSA.

Participants currently receiving treatment for OSA with continuous positive airway pressure (CPAP), answered the BQ based on symptoms prior to the CPAP treatment. We scored the BQ according to the scoring manual, and two or more positive category scores were considered as high risk of OSA [29, 31].

### Statistical analyses

Descriptive statistics are presented as frequencies (n) with percentages (%) for proportions, or means with standard deviation (SD) for continuous variables. Independent sample t-tests with 95% confidence intervals (CI) and p-values were used to compare means between groups. Score 95% CI and continuity corrected chi-squared tests were used for comparing proportions (applying the “prop. test” R function). CI for proportions was found using Exact Binominal Tests (applying the “binom.test” R function). Logistic regression was used to analyze for potential predictors of OSA. The predictors were chosen based on our clinical experience with achondroplasia, and literature reports on average-statured individuals [9–11]. Logistic regression results are reported as odds ratios (ORs) with 95% CI. Statistical significance was set to p < 0.05 (two-sided). Statistical analyzes was performed using the Statistical Package for Social Sciences (SPSS) version 25 (IBM Corp., Armonk, New York), and R version 4.0.

## Results

### Study population and clinical characteristics

Forty-nine of the 50 participants in The Norwegian Adult Achondroplasia Study were included in this study (27 men and 22 women). One participant was not able to conduct the sleep registration due to impaired health, and was therefore excluded. Mean age of the study population was 40 years, ranging from 16 to 87 years. Table 1 details the characteristics of the study population. All participants had genetically confirmed achondroplasia [24]. Of those with a preexisting diagnosis of OSA (n = 15), as diagnosed by polysomnography, the previous sleep records were not accessible for three participants. These three had an overnight sleep registration (with the NOX T3) during the stay, confirming their diagnosis of OSA.

**Table 1:** Characteristics of adult participants with achondroplasia

Variables	All (n = 49)	Men (n = 27)	Women (n = 22)
Age, years, mean (SD)	39.8 (18.3)	42.7 (20.0)	36.2 (15.8)
Single/living alone, n (%)	18 (37)	10 (37)	8 (36)
Working or student, n (%)	28 (57)	13 (48)	15 (68)
Obstructive sleep apnea, n (%)	29 (59)	19 (70)	10 (45)
Hypertension, n (%) ^a^	17 (35)	14 (52)	3 (14)
Current smoking, n (%)	5 (10)	4 (15)	1 (5)
History of adenoidectomy, n (%)	23 (47)	13 (48)	10 (46)
History of tonsillectomy, n (%)	17 (35)	11 (41)	6 (27)

	Mean (SD)	Mean (SD)	Mean (SD)
Height, cm	132.5 (9.3)	135.4 (9.5)	129.1 (7.8)
Weight, kg	58.9 (13.9)	62.4 (15.8)	54.5 (9.8)
Body mass index, kg/m^2^	33.4 (6.7)	34.0 (7.6)	32.7 (5.5)
Waist circumference, cm	87.2 (14.6)	91.3 (16.4)	81.5 (10.0)
Systolic blood pressure, mm Hg	121.8 (15.6)	125.3 (16.5)	117.4 (14.4)
Diastolic blood pressure, mm Hg	74.8 (11.0)	76.6 (11.4)	72.6 (10.4)

^a^Hypertension was defined as systolic blood pressure ≥ 140 mm Hg, diastolic blood pressure ≥ 90 mm Hg, or antihypertensive drug treatment

### Obstructive sleep apnea

**Fig. 1:**
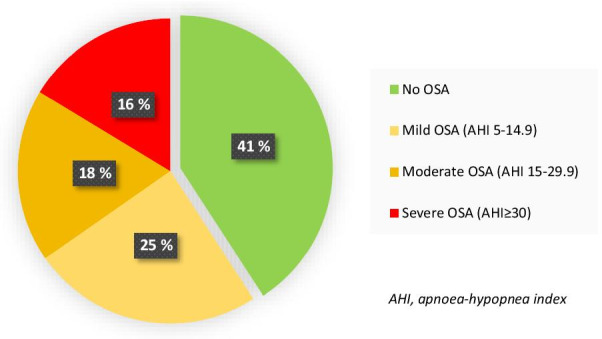
Prevalence and severity of obstructive sleep apnea (OSA) in Norwegian adults with achondroplasia

OSA was found in 59% (29/49; 95% CI 44% to 73%) of the participants, including 70% (19/27) of the men and 45% (10/22) of the women. All but one had been diagnosed in adulthood, and 48% (14/29; 95% CI 29% to 67%) were previously undiagnosed with OSA. Of all participants, 35% (17/49) had moderate to severe OSA (Fig. 1). In the OSA group, the majority (27/29) had mainly obstructive sleep apneas, while two participants had mainly central apneas. Forty-eight percent (14/29) had CPAP treatment.

### Berlin questionnaire

All participants completed the BQ. Snoring was reported by 86% of the participants (42/49), loud snoring (louder than talking) by 37% (18/49), excessive daytime sleepiness by 59% (29/49), unrested sleep by 49% (24/49), and observed breathing stops by 24% (12/49).

Of all participants, 69% (34/49) had a total BQ score of ≥ 2, indicating high risk of OSA. The sensitivity of the BQ to identify those with OSA was 86% (25/29, 95% CI 68 to 96%), while the specificity was 55% (11/20; 95% CI 32% to 77%). The positive predictive value (PPV) was 74%, while the negative predictive value (NPV) was 73%.

### Variables predictive of OSA

Mean (SD) BMI was 35.8 (6.9) kg/m^2^ in the OSA group, compared to 30.1 (4.7) kg/m^2^ in the non-OSA group. Mean difference was 5.7 kg/m^2^ (95% CI 2.1 to 9.3; *p* = 0.002). Hypertension was found in 48% (14/29) of the OSA group, compared to 15% (3/20) in the non-OSA group (OR 5.3; 95% CI 1.3 to 22.0; *p* = 0.02). A positive score on each of the other following single component BQ items were predictive of the presence of OSA: loud snoring; observed nocturnal breathing stop; unrested sleep; excessive daytime sleepiness; age over 40 years; and BMI over 30 kg/m^2^ (Table 2).

**Table 2:** Predictors of obstructive sleep apnea (OSA) in adults with achondroplasia

Variables	OSA (n = 29)	No OSA (n = 20)	Difference	OR (95% CI)	*P* value
Age > 40 years, %	66	25	41	5.7 (1.6 to 20.3)	0.007
Male gender, %	66	40	26	2.9 (0.9 to 9.3)	0.08
Current smoking, %	7	15	− 8	0.4 (0.1 to 2.8)	0.37
History of adenoidectomy, %	48	45	3	1.1 (0.4 to 3.6)	0.82
History of tonsillectomy, %	41	25	16	2.1 (0.6 to 7.4)	0.24
**Berlin Questionnaire (BQ)**					
Total BQ score ≥ 2 (high risk), %	86	45	41	7.6 (1.9 to 30.2)	0.004
**BQ category 1 (snoring)**					
Snoring, %	90	80	10	2.2 (0.4 to 11.0)	0.35
Loud snoring, %	52	15	37	6.1 (1.5 to 25.3)	0.01
Frequent snoring, %	76	55	21	2.6 (0.8 to 8.7)	0.13
Snoring bothering other, %	69	55	14	1.8 (0.6 to 5.9)	0.56
**BQ category 2 (sleep)**					
Observed breathing stops, %	38	5	33	11.6 (1.4 to 99.3)	0.03
Unrested sleep, %	66	25	41	5.7 (1.6 to 20.3)	0.007
Excessive daytime sleepiness, %	72	40	32	3.9 (1.2 to 13.2)	0.03
Fallen asleep while driving, %	24	10	14	2.9 (0.5 to 15.5)	0.22
**BQ Category 3**					
Hypertension, %^a^	48	15	33	5.3 (1.3 to 22.0)	0.02
Body mass index > 30 kg/m^2^, %	83	50	33	4.8 (1.3 to 17.7)	0.02

^a^ Hypertension was defined as systolic blood pressure ≥ 140 mm Hg, diastolic blood pressure ≥ 90 mm Hg, or antihypertensive drug treatment

### Comparison of participants with OSA, with or without a preexisting diagnosis of OSA

In the OSA group (n = 29), we compared those with a preexisting diagnosis of OSA (n = 15) with those who were diagnosed with OSA during the study (n = 14) (Table 3). The majority of participants with a preexisting diagnosis of OSA were men (87% versus 43%; *p* = 0.04), and had significantly higher prevalence of observed breathing stops (67% versus 7%; *p* = 0.004). There were no major differences between the two groups regarding the other clinical variables or BQ scores, or the total BQ score (Table 3).

**Table 3:** Comparison of participants with obstructive sleep apnea (OSA), with or without a pre-existing diagnosis of OSA

Variables	Pre-existing OSA diagnosis		Difference (95% CI)	*P* Value
	Yes (n = 15)	No (n = 14)		
Age > 40 years, %	80	50	30 (− 10 to 70)	0.19
Male gender, %	87	43	44 (6 to 82)	0.04
Current smoking, %	7	7	0 (− 19 to 18)	1.0
Moderate to severe OSA (AHI ≥ 15), %	67	50	17 (− 26 to 59)	0.59
**Berlin Questionnaire (BQ)**				
Total BQ score ≥ 2 (high risk), %	93	79	14 (− 17 to 47)	0.54
**BQ category 1 (snoring)**				
Snoring, %	87	93	− 6 (− 34 to 22)	1.0
Loud snoring, %	53	50	3 (− 36 to 43)	1.0
Frequent snoring, %	80	71	9 (− 29 to 47)	0.92
Snoring bothering others, %	80	57	23 (− 17 to 63)	0.35
**BQ category 2 (sleep)**				
Observed breathing stops, %	67	7	60 (25 to 94)	0.004
Unrested sleep, %	73	57	16 (− 25 to 57)	0.60
Excessive daytime sleepiness, %	67	79	− 12 (− 42 to 31)	1.0
Fallen asleep while driving, %	27	21	6 (− 31 to 42)	1.0
**BQ category 3**				
Hypertension, %^a^	47	50	0 (− 43 to 36)	1.0
Body mass index > 30 kg/m^2^, %	87	79	8 (− 26 to 43)	0.93

^a^Hypertension was defined as systolic blood pressure ≥ 140 mm Hg, diastolic blood pressure ≥ 90 mm Hg, or antihypertensive drug treatment

## Discussion

In this population-based study, we found a high prevalence of OSA (59%) in Norwegian adults with achondroplasia. Of those with OSA, 59% had moderate to severe OSA (AHI ≥ 15), and almost half were previously undiagnosed. Excessive daytime sleepiness, unrested sleep, loud snoring, observed breathing stops, age over 40 years, hypertension, and BMI over 30 kg/m^2^, were predictive of OSA in the study sample.

Individuals with craniofacial syndromes are at high risk of sleep-related breathing disorders, where OSA is the most common [32–34]. However, few studies have investigated OSA in adult skeletal dysplasia populations, and we are not aware of previous population-based studies on OSA in adults with achondroplasia [17].

The high prevalence of OSA in our study is consistent with previous studies conducted in children with achondroplasia, with a reported OSA prevalence of 50–80% [13–15]. Furthermore, our findings are consistent with a recently published US study, having included 114 children and adults with achondroplasia, reporting of an overall OSA prevalence of 69% [35].

The pathophysiology of OSA in achondroplasia is complex and multifactorial, and not fully understood [19]. In children, the high prevalence of OSA is thought to be caused by a combination of the abnormal craniofacial anatomy, including midface hypoplasia, depressed nasal bridge and mandibular prognathism, adenoid and tonsil hypertrophy, and airway muscles hypotonia [13, 19–21, 33, 34, 36]. In our study, almost half of the participants had a history of adenoidectomy, and one-third had undergone tonsillectomy. However, a history of adenoidectomy and/or tonsillectomy in childhood did not appear to strongly influence on the presence of OSA in our adult study population. This is consistent with previous studies having demonstrated persistent OSA in children with achondroplasia after having undergone adenotonsillectomy [18, 20, 21]. Similar findings have also been reported in other craniofacial syndromes [32, 37]. Despite having had adenotonsillectomy in childhood, the abnormal craniofacial anatomy persists in adults with achondroplasia, predisposing for OSA.

In addition, individuals with achondroplasia have a propensity for obesity [5–7]. Increased BMI is a well-known risk factor of OSA in the average-statured population [12, 38]. About 70% of the participants in our study had BMI over 30 kg/m^2^, which might contribute to the observed high prevalence of OSA. Having BMI over 30 kg/m^2^ was significantly associated with the presence of OSA (OR 4.8). The findings underline the importance of preventing excessive weight gain in achondroplasia by establishing healthy dietary and physical activity habits early in life [22].

The prevalence of hypertension was significantly higher in the OSA group (48%) compared to the non-OSA group (15%). There is strong evidence from studies on average-statured individuals that OSA might play a causal role in the development of hypertension [11, 12, 39]. Moreover, OSA is associated with increased risk of cardiovascular disease, stroke and premature mortality [12, 39]. An increased risk of premature cardiovascular mortality, and a high prevalence of hypertension, have been reported in adults with achondroplasia [40, 41]. Our findings suggest that in the presence of hypertension in an individual with achondroplasia, additional symptoms indicative of OSA should be assessed, with a low threshold for referral for an overnight sleep registration to assess presence and severity of OSA, and to treat accordingly (i.e. with devices that deliver continuous positive airways pressure during sleep) [42].

In this study, we used the BQ in order to standardize the interview questions and assessment of OSA-related symptoms. We are not aware of specific OSA questionnaires used in or validated for achondroplasia. According to the scoring manual, a total BQ score of ≥ 2 indicates a high risk of OSA [29, 31]. However, use of the BQ and other similar questionnaires for screening of OSA is controversial, despite being widely used [8]. Recent studies have reported a low sensitivity (about 76%) and specificity (about 45%) of the BQ for detecting OSA in the general population, resulting in a large number of false negative results [8, 30]. In our study, the sensitivity and specificity were somewhat higher, 86% and 55% respectively, giving a PPV of 74% and NPV of 73%. The American Academy of Sleep Medicine recommends the BQ and similar clinical tools and prediction algorithms not to be used to diagnose OSA in adults, in the absence of polysomnography or home sleep monitoring [8]. These recommendations also seem appropriate for achondroplasia.

The single BQ items significantly predictive of the presence of OSA in our study (excessive daytime sleepiness, unrested sleep, loud snoring, observed nocturnal breathing stops, hypertension, and BMI over 30 kg/m^2^) are similar to what has been reported in average-statured populations [9, 10].

There were no considerable differences between those with a preexisting diagnosis of OSA and those without, except for the variables of gender (more men), and prevalence of observed breathing stops (higher prevalence) in those with a preexisting OSA diagnosis. A possible explanation might be that patients reporting observed breathing stops are more likely to be recognized as suspect of having OSA, and referred for a sleep registration, while other symptoms or signs of OSA are more subtle. Studies on average-statured populations have found that women with symptoms of sleep-disordered breathing were less likely to be diagnosed and treated for sleep apnea than men, although the consequences of the disease appear to be similar, or worse [11, 43]. Overall, the findings underline the importance of having a low threshold for screening for OSA in adults with achondroplasia, in men and women, in the presence of any symptoms or signs suggestive of OSA (Table 4).

**Table 4:** Recommendations for clinical practice^a^

1. Follow-up of adults with achondroplasia should include systematic assessment of symptoms and signs of OSA
2. OSA should be suspected in the presence of excessive daytime sleepiness in combination with at least one of the following
• Habitual, loud snoring (louder than talking)
• Observed nocturnal breathing stops, choking or gasping
• Diagnosed hypertension
• Body mass index > 30 kg/m^2^
3. If OSA is suspected, an overnight sleep registration should be performed, preferably by polysomnography, or with an adequate home-based portable sleep monitor
4. If a single home-based sleep test is negative in symptomatic individuals, polysomnography should be performed
5. Referral to a respiratory/sleep physician should be considered for appropriate management and follow-up of OSA if present

^a^The recommendations are based on the Clinical Practice Guideline for Diagnostic Testing for Adult Obstructive Sleep Apnea, provided by the American Academy of Sleep Medicine ^8^, and modified for adults with achondroplasia according to our clinical experience and the findings in the present study

### Strengths and limitations

A major strength of this study is the population-based study sample, with genetically confirmed achondroplasia in all participants. The clinical approach, no missing data, and all participants having undergone an objective sleep apnea investigation, are other notable strengths.

There are also limitations to this study. First, according to the American Academy of Sleep Medicine, polysomnography is the recommended standard for diagnosing OSA in average-statured adults [8]. Home sleep monitoring could be an alternative in individuals presenting with increased risk of moderate to severe OSA, and without other medical complications [8]. Home sleep monitoring is less sensitive than polysomnography in the detection of OSA and may therefore give a false negative result [8]. Hence, there is a risk that we may have overlooked some of those with mild OSA, resulting in underestimation of the prevalence of OSA in our study sample. However, with manual scoring of the home sleep records by trained personnel, the sensitivity and specificity of this method have been reported to be high compared to polysomnography [44, 45].

Second, those with a preexisting diagnosis of OSA, and currently receiving CPAP treatment, answered the BQ based on symptoms prior to the CPAP treatment. This might give a risk of recall bias, most likely resulting in underreporting of the symptoms of OSA.

Finally, the exact prevalence of achondroplasia within Norway is uncertain. We have estimated the expected population of adults (16 years or older) with achondroplasia in Norway to be between 66 and 101 adults [24]. As this study included somewhat fewer participants, there is a risk of selection bias that could have affected these outcomes. Our findings should therefore be confirmed by other larger studies in different adult populations with achondroplasia, and preferably using polysomnography to assess OSA.

### Implications for clinical practice

OSA appears to have greater prevalence in adults with achondroplasia as compared to the general population. We suggest that regular assessment of symptoms and signs of OSA should be part of standard health care in adults with achondroplasia, as it currently is in children with this condition [22]. Moreover, clinicians should have a low threshold for referral to a respiratory (sleep) physician and for an overnight sleep registration if symptoms or signs suggestive of OSA are present in adults with achondroplasia (Table 4).

## Conclusion

OSA was highly prevalent in this population-based study of Norwegian adults with achondroplasia, which we believe is representative of this population worldwide [24]. Excessive daytime sleepiness, unrested sleep, loud snoring, observed nocturnal breathing stops, age over 40 years, hypertension, and BMI over 30 kg/m^2^, were predictive of the presence of OSA. We propose that follow-up of adults with achondroplasia should include systematic assessment for symptoms and signs of OSA, with a low threshold for conducting an overnight sleep registration if findings suggestive of OSA are present. These data will assist in establishing baselines with regard to the prevalence and severity of OSA in adults with achondroplasia, which are timely given the recent emergence of therapies being trialed in children with achondroplasia [46] that might improve these complications in the future. The pathophysiology of OSA in achondroplasia is not yet fully understood, and the contribution of abnormal craniofacial skeletal morphology, soft tissue structures, and other causal factors to its etiology, will require further study.

## Data Availability

De-identified individual participant data are available from the corresponding author on reasonable request.

## Declarations

### Clinical trial registration

ClinicalTrials.gov identifier NCT03780153.
